# Biomarkers of neurodegeneration in schizophrenia: systematic review and meta-analysis

**DOI:** 10.1136/bmjment-2024-301017

**Published:** 2024-05-24

**Authors:** Jack Christopher Wilson, Kathy Y Liu, Katherine Jones, Jansher Mahmood, Utkarsh Arya, Rob Howard

**Affiliations:** 1 University College London, London, UK; 2 Division of Psychiatry, University College London, London, UK; 3 Camden and Islington NHS Foundation Trust, London, London, UK; 4 University College London Medical School, London, UK; 5 Sussex Partnership NHS Foundation Trust, Worthing, UK

**Keywords:** Schizophrenia & psychotic disorders

## Abstract

**Question:**

Does neurodegenerative disease underlie the increased rate of dementia observed in older people with schizophrenia? Several studies have reported a higher prevalence of dementia in people with schizophrenia compared with the general population. This may reflect a higher risk of developing neurodegenerative diseases such as vascular dementia or Alzheimer’s disease (AD). Alternatively, this may reflect non-pathological, age-related cognitive decline in a population with low cognitive reserve.

**Study selection and analysis:**

We reviewed papers that compared postmortem findings, hippocampal MRI volume or cerebrospinal fluid (CSF) markers of AD, between patients with schizophrenia with evidence of cognitive impairment (age ≥45 years) with controls. We subsequently performed a meta-analysis of postmortem studies that compared amyloid-β plaques (APs) or neurofibrillary tangles (NFTs) in cognitively impaired patients with schizophrenia to normal controls or an AD group.

**Findings:**

No studies found a significant increase of APs or NFTs in cognitively impaired patients with schizophrenia compared with controls. All postmortem studies that compared APs or NFTs in patients with schizophrenia to an AD group found significantly more APs or NFTs in AD. No studies found a significant differences in CSF total tau or phosphorylated tau between patients with schizophrenia and controls. The two studies which compared CSF Aβ42 between patients with schizophrenia and controls found significantly decreased CSF Aβ42 in schizophrenia compared with controls. Hippocampal volume findings were mixed.

**Conclusions:**

Studies have not found higher rates of AD-related pathology in cognitively impaired individuals with schizophrenia compared with controls. Higher rates of dementia identified in population studies may reflect a lack of specificity in clinical diagnostic tools used to diagnose dementia.

**WHAT IS ALREADY KNOWN ON THIS TOPIC**

- Several large epidemiological studies have identified apparently higher rates of Alzheimer’s disease (AD) and dementia in the schizophrenia population. No previous study has systematically reviewed whether established markers of neurodegeneration are present in cognitively impaired people with schizophrenia.

**WHAT THIS STUDY ADDS**

- We found an absence of excess of such markers in cognitively impaired older individuals with schizophrenia, casting doubt on whether common neurodegenerative conditions, such as AD, truly underlie reported higher rates of dementia in the schizophrenia population.

**HOW THIS STUDY MIGHT AFFECT RESEARCH, PRACTICE OR POLICY**

- Higher rates of dementia in schizophrenia may be mainly related to a lack of specificity in dementia diagnostic tests and the possibility that age-related cognitive decline in a group with low cognitive reserve can cause a diagnostic threshold for dementia to be crossed earlier.

## Introduction

Several studies over recent years have reported increased risk of dementia in people with schizophrenia compared with the general population and those with other psychiatric diagnoses.^1–5^ However, the mechanisms behind this are unclear, and it is not known whether specific neurodegenerative disease processes are involved. The hallmark of dementia is *progressive* cognitive decline; that is, worsening and irreversible symptoms related to neurodegenerative disease processes, manifesting over months to years, and beyond what might be expected due to ageing. Cognitive impairment in schizophrenia is well established and observed throughout the course of the illness and, despite heterogeneous trajectories, is believed to remain relatively stable in most cases, notwithstanding the short follow-up of several studies.^6–8^ There is a paucity of longitudinal cognitive data in older people with chronic schizophrenia, which may also be influenced by the effects of institutionalisation.^9^ It would be important to determine whether an observed increased risk of dementia can be accounted for by identified neurodegenerative disease processes, otherwise researchers and clinicians risk misclassifying cognitive symptoms of schizophrenia in older people as comorbid dementia.

There are plausible reasons why schizophrenia may truly be associated with higher rates of dementia. Known dementia risk factors, such as smoking, obesity and diabetes, are higher in this population,^10 11^ and average educational attainment and consequent cognitive reserve are lower.^12^ There may also be overlapping genetic risk for schizophrenia and neurodegenerative disorders.^13–15^

Alternatively, apparent higher dementia rates in schizophrenia may be an artefact of assessing older individuals who are experiencing age-related cognitive decline,^12^ but, due to low cognitive reserve, have crossed a clinical threshold that warrants a dementia diagnosis. Lower scores on cognitive testing are a key component of diagnosing dementia; patients with schizophrenia may also perform poorly on these tests due to deficits in attention, working memory, verbal fluency and executive functioning, which are inherent to schizophrenia, as well as the effects of associated drug treatment,^16 17^ and institutionalisation,^9^ rather than underlying neurodegeneration.^18^ Likewise, independent functioning and activities of daily living are often impaired in patients with schizophrenia at baseline. This further complicates diagnosing dementia, as loss of function is another key component of the diagnosis.

Evidence of neurodegeneration, for example, Alzheimer’s disease (AD) neuropathology, in older patients with schizophrenia with cognitive impairment or a diagnosis of dementia would support the proposal that increased neurodegeneration underlies a higher risk of developing dementia. Alternatively, if rates of neurodegeneration markers in this group mirror those in the general population, the observed increased dementia rates in the schizophrenia population likely have an alternative explanation. Although a large postmortem neuropathological study of 100 cases found no postmortem evidence of AD in cognitively impaired older individuals with schizophrenia,^19^ there has been no systematic assessment of the findings and quality of those studies that have investigated any (including histopathological, fluid and neuroimaging) markers of common neurodegenerative conditions in this population.

We aimed to review the literature on neuropathological and neurodegeneration biomarker studies in older people with schizophrenia, who showed evidence of cognitive impairment or had a clinical diagnosis of dementia, to investigate whether neurodegenerative disease processes could underlie the increased rate of dementia observed in this population. A secondary aim was to synthesise the findings of studies in a meta-analysis if the relevant data were available.

## Methods

Our review was structured based on the principles set out in the Preferred Reporting Items for Systematic Reviews and Meta-Analyses statement 2020.

### Literature search

Online literature databases (PubMed (from 1964), Web of Science (from 1900) and Embase (from 1974)) were initially searched up to 29 October 2021 and subsequently updated on 31 January 2023, using the search term “(schizophreni* OR hebephrenia OR 'dementia praecox') AND (dementia OR neurodegenerati* OR Alzheimer* OR 'cognitive impairment' OR 'cognitive dysfunction' OR 'cognitive decline' OR 'cognition disorders' OR 'mild cognitive impairment') AND (neuropatholog* OR patholog* OR postmortem OR postmortem OR autopsy OR amyloid OR tau OR 'neurofilament light chain' OR hippocamp*) AND (older OR elder* OR 'old age' OR 'geriatric' OR aging OR ageing OR 'increasing age' OR 'middle age*' OR 'middle-age* OR aged OR mid-life)”.

### Inclusion/exclusion criteria

Studies were included if they were published, peer-reviewed, observational articles in English on human subjects aged ≥45 years with a diagnosis of schizophrenia. In addition, subjects had either a comorbid diagnosis of dementia or evidence of cognitive impairment on quantitative neuropsychological tests. Age 45 was chosen rather than the more standard cut-off of 65 due to evidence that cognitive impairment and dementia occur earlier in people with schizophrenia. The lower threshold was set so that we did not unintentionally miss out these cases. Studies were included if they investigated neurodegeneration markers and included a comparison group (ie, was a case–control study). Neurodegeneration markers were defined as one or more of the following:

- Amyloid-β plaques (APs), abnormal tau/neurofibrillary tangles (NFTs), cerebral amyloid angiopathy, Transactive response DNA binding protein of 43 kDa (TDP-43, hippocampal sclerosis, Lewy bodies or vascular pathologies such as infarcts, atherosclerosis and arteriolar sclerosis, identified in postmortem analyses
- Any other marker not mentioned above, which was directly compared between individuals with schizophrenia versus a neurodegenerative condition, including AD, Parkinson’s disease (PD), Lewy body dementia (DLB) and vascular dementia
- Amyloid or tau, quantified using positron emission tomography (PET), cerebrospinal fluid (CSF) or plasma measures
- Neurofilament light chain analysis in CSF
- Hippocampal volume analysis using MRI

We excluded postmortem studies that only assessed changes in neurotransmitter systems, or neuropathological changes not associated with neurodegeneration/neurodegenerative disorders (unless they were direct comparisons between schizophrenia and a neurodegenerative group, as above). We also excluded studies that examined other psychotic illnesses, such as bipolar disorder or schizophrenia spectrum disorders (ie, schizotypal personality disorder, schizoaffective disorder, schizophreniform disorder), and very late-onset schizophrenia-like psychosis, and studies that grouped patients with schizophrenia with these disorders if it was not possible to isolate results for the patients with schizophrenia. Clinical trials, reviews, case studies, conference abstracts or book chapters were also excluded.

### Data extraction

Two authors (out of UA, JCW, KJ, JM and KYL) independently screened all papers for inclusion based on their titles and abstracts. Two authors (out of UA, JCW, KJ and JM) then independently assessed the remaining full-text articles for eligibility and performed data extraction on the included papers. For each paper, data were sought on what pathology was investigated, pathological findings (including where relevant APs/NFT concentration, descriptive postmortem findings, mean hippocampal volume, levels of CSF markers), location of pathology, what groups were compared, numbers of participants in each group, total number of enrolled participants, participant average age in each group, percentage female participants in each group, which neuropsychological tests were used, average scores in neuropsychological tests for each group, mean years of education in each group and whether study authors concluded that cognitive decline/dementia observed in participants with schizophrenia was primarily the result of neuropathological changes. Any discrepancies were discussed and re-examined by the two authors and a third author was consulted (KYL) if required. An assessment of the quality of included studies was also conducted using a modified version of the NIH Quality Assessment Tool for Observational Cohort and cross-sectional studies (two questions were omitted due to lack of relevance to our study: *For exposures that can vary in amount or level, did the study examine different levels of the exposure as related to the outcome* and *was the exposure assessed more than once over time?*) Studies were given a score of between 0 and 12, with 0–4 classed as low, 5–8 classed as medium and 9–12 classed as high.

### Meta-analysis

Following our systematic review of the literature, we were able to perform a meta-analysis on studies that reported data on AD pathology. Suitable studies compared results for either the density of APs or NFTs (or both) between cognitively impaired patients with schizophrenia and normal older controls or an AD group (or both). Studies that provided quantitative results with either an identifiable SD or SE were included. Where studies provided AP or NFT counts from different areas of the brain, we preferentially selected results from the hippocampus (present in the majority of studies), followed by the neocortex, then subcortical structures.

Suitable studies were included in a random-effects meta-analysis model to compute pooled weighted effect sizes and 95% CIs across studies using the ‘dmetar’ package (V.0.0.9000) in R V.4.3.1. Effect sizes (Hedge’s G) were the between-group standardised mean differences (Cohen’s d) for APs or NFTs in schizophrenia versus normal older controls and schizophrenia versus AD dementia, represented as forest plots. Study heterogeneity was measured using the I^2^ statistic.

## Results

### Identification and characteristics of included studies

**Figure 1:**
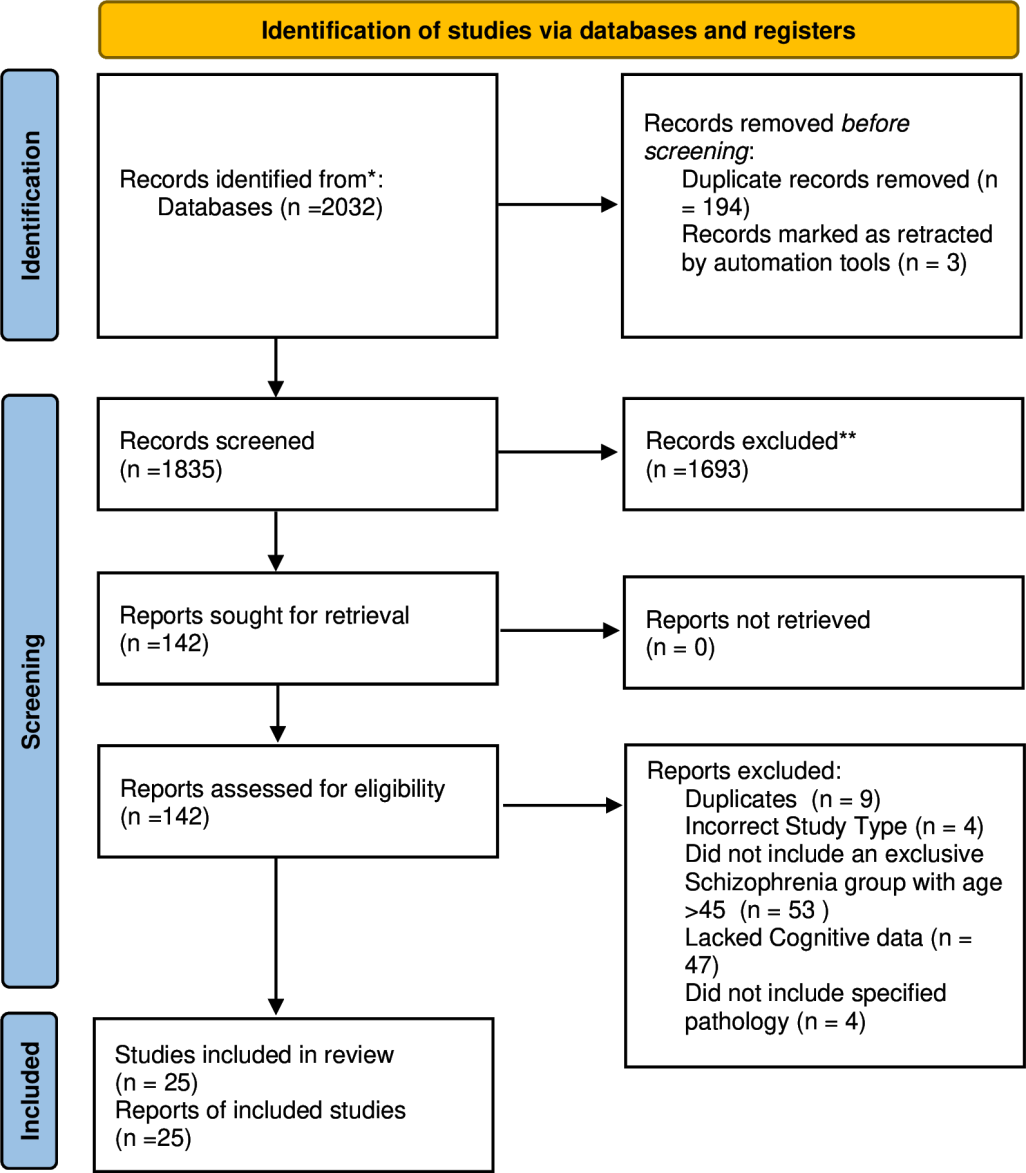
Preferred Reporting Items for Systematic Reviews and Meta-Analyses 2020 flow diagram for new systematic reviews which included searches of databases and registers only.

Initial literature searches identified 1696 potential studies, of which 24 relevant studies were included after screening (figure 1). During the updated literature search on 31 January 2023 a further 336 potential studies were screened, of which one additional relevant study was included. The most common assessment methods employed by included studies (17/25, 68%) were postmortem neuropathological changes, followed by hippocampal MRI volume studies (5/25, 20%), and CSF studies of AD biomarkers (3/25, 12%) (online supplemental table 1). Postmortem studies mainly investigated AD-related pathology, that is, either amyloid plaques, NFTs or both, although several postmortem studies also investigated a range of other markers, including histopathological changes (eg, features of Parkinson’s and Lewy body disease, vascular pathology), protein expression and the presence of various peptides and ions. Where authors had included statistical comparisons between groups, we included these in online supplemental table 1. For relevant descriptive findings, we have included these in the notes to online supplemental table 1.

10.1136/bmjment-2024-301017.supp2 Supplementary data

### Neurodegeneration marker findings

Out of the 15 studies that investigated AD-related pathology on postmortem examination, no studies found significant differences between cognitively impaired patients with schizophrenia and normal control groups. Nine postmortem studies compared APs or NFTs in people with schizophrenia to an AD control group; all of these studies found significantly more APs or NFTs in the AD group compared with the schizophrenia group (online supplemental table 1). Some authors included other AD-related markers, including acetylcholinesterase activity,^20^ Alz-50 immunoreactivity (a marker for tau)^21^ and the number of neurons that stained positive for the presence of tau.^22^ In each of these studies, the cognitively impaired schizophrenia group did not differ significantly from the normal control group and showed significantly lower levels of the AD pathology compared with an AD control group.

Decreases in CSF concentrations of amyloid-beta 42 (Aβ42) and elevations in total tau and phosphorylated tau are used as biomarkers for AD.^23^ Two studies that measured CSF Aβ42 levels found significantly lower levels of Aβ42 in patients with schizophrenia compared with normal controls (online supplemental table 1). These studies found the AD comparison groups had significantly lower Aβ42 than both patients with schizophrenia and controls.^24 25^ Patients with AD also had significantly elevated levels of T-tau and P-tau compared with patients with schizophrenia. No significant differences in CSF T-tau or P-tau levels were found between patients with schizophrenia and normal controls in any of the CSF studies.^26^

Studies that investigated hippocampal MRI volume differences have reported mixed findings. One study comparing treatment resistant patients with schizophrenia (TRS) and non-treatment resistant patients with schizophrenia (NTRS) found significantly smaller hippocampal volumes in TRS compared with normal controls but no difference between NTRS and normal controls.^27^ A further study did not find any significant difference in hippocampal volume between patients with schizophrenia and normal controls but did note that there was a steeper decline in hippocampal volume as a function of age in the schizophrenia group.^28^ One study found smaller left-sided hippocampal–amygdala volumes for schizophrenia versus normal controls.^29^ Rivas *et al* compared patients with schizophrenia who met the fifth edition of the Diagnostic and Statistical Manual of Mental Disorders (DSM V) criteria for dementia with patients with schizophrenia who did not, and normal controls. The schizophrenia group with dementia had significantly lower mean whole hippocampal volumes compared with the normal control group, while the patients with schizophrenia without cognitive impairment showed no significant difference compared with normal controls. Differences in whole hippocampal volume between the schizophrenia group with dementia and the schizophrenia group without dementia were not significant.^30^ Prestia *et al* found that cognitively impaired patients with schizophrenia had similar hippocampal volumes to patients with AD, and significantly lower volumes than controls.^31^

No studies that used amyloid or tau serum markers met the criteria for inclusion in this review.

### Meta-analysis findings

**Figure 2:**
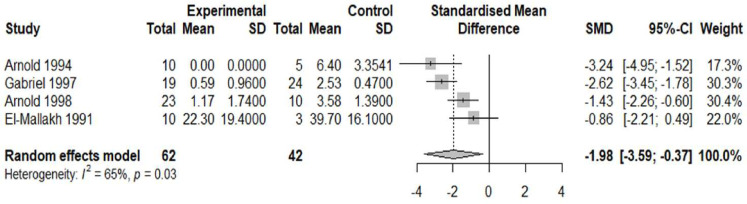
Density of amyloid plaques in schizophrenia versus Alzheimer’s. Forest plot showing the effect size (Hedge’s G) and the pooled effect size for studies comparing amyloid-β plaques in the schizophrenia group (‘experimental’) versus Alzheimer’s disease (‘controls’). ‘Total’ denotes the sample size and SMD denotes standardised mean difference.

**Figure 3:**
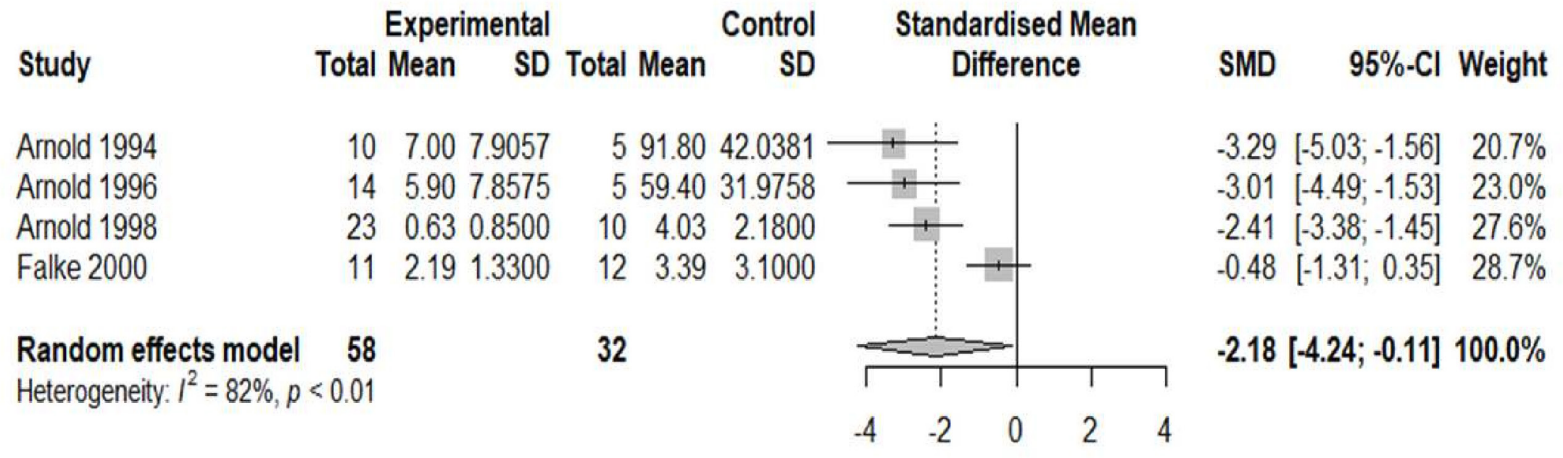
Density of neurofibrillary tangles (NFTs) in schizophrenia versus Alzheimer’s. Forest plot showing the effect size (Hedge’s G) and the pooled effect size for studies comparing NFTs in the schizophrenia group (‘experimental’) versus AD (‘control’). ‘Total’ denotes the sample size and SMD denotes standardised mean difference.

**Figure 4:**
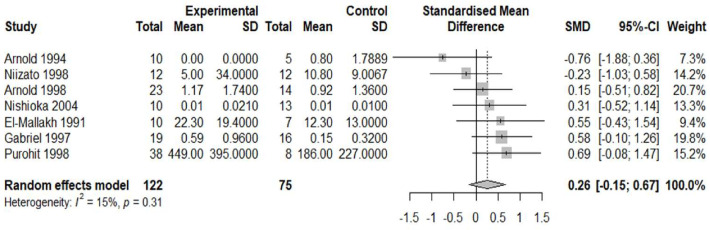
Density of amyloid plaques in schizophrenia versus controls. Forest plot showing the effect size (Hedge’s G) and the pooled effect size for studies comparing amyloid-β plaques in the schizophrenia group (‘experimental’) versus normal controls (‘controls’). ‘Total’ denotes the sample size and SMD denotes standardised mean difference.

**Figure 5:**
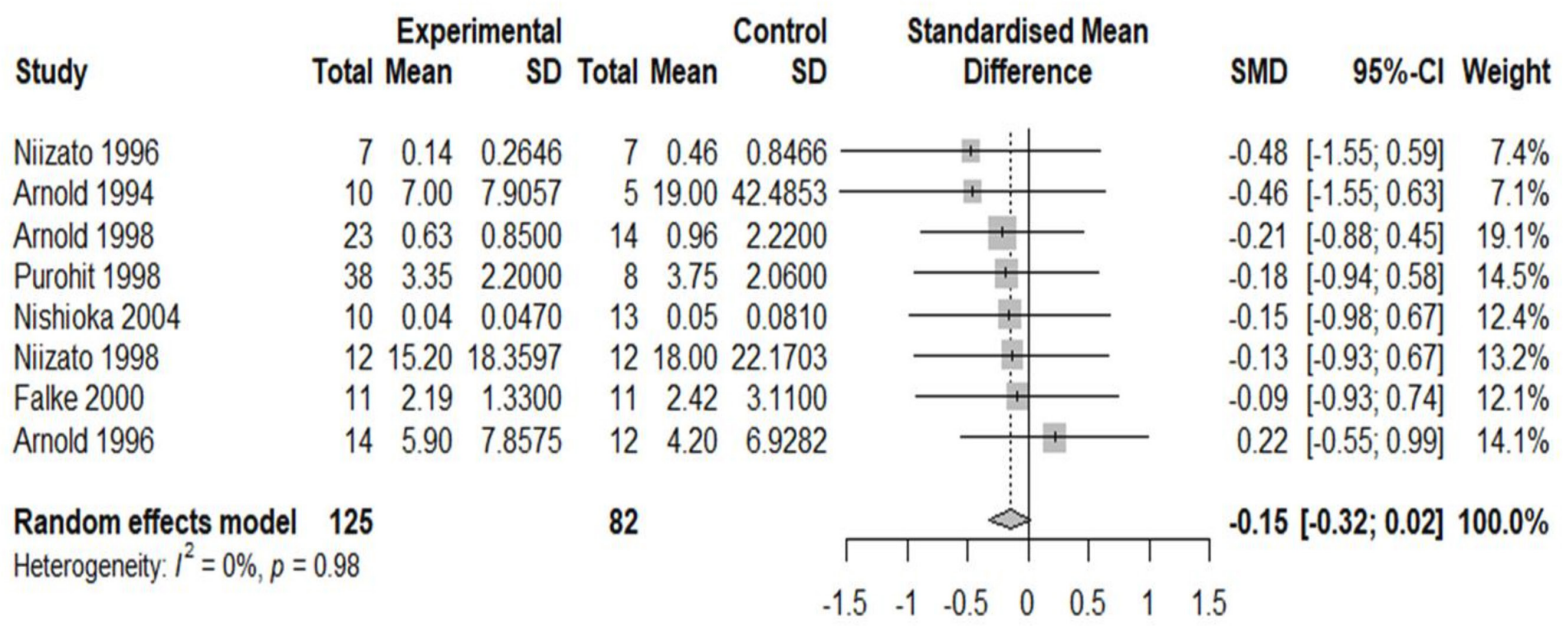
Density of neurofibrillary tangles (NFTs) in schizophrenia versus controls. Forest plot showing the effect size (Hedge’s G) and the pooled effect size for studies comparing NFTs in the schizophrenia group (‘experimental’) versus normal controls (‘control’). ‘Total’ denotes the sample size and SMD denotes standardised mean difference.

10 studies were deemed suitable for meta-analysis. Details of why studies were excluded from the meta-analysis are presented in online supplemental table 1. The pooled effect size (standardised mean difference or Cohen’s d) for included studies comparing APs in the schizophrenia versus AD group was −1.98 (95% CI −3.59 to −0.37) (figure 2) and the pooled effect size for included studies comparing NFTs in the schizophrenia group versus AD was −2.18 (95% CI−4.24 to −0.11) (figure 3). Thus, the pooled effect size was significantly lower in schizophrenia versus patients with AD for both APs and NFTs. There was no overall significant difference in the number/density of APs (pooled effect size 0.26, 95% CI −0.15 to 0.67) (figure 4) or NFTs (pooled effect size −0.15, 95% CI −0.32 to 0.02) (figure 5) between the schizophrenia and normal control groups.

## Discussion

Impaired cognition is a core symptom of schizophrenia that precedes the onset of psychosis, is a key determinant of poor functioning and is the most resistant to treatment. It remains unclear the extent to which cognition worsens subsequent to diagnosis.^32 33^ Recent studies have suggested that there is an additional modest decline in cognitive functioning in later life in people with schizophrenia (though this decline is heterogeneous, and detection is dependent on the cognitive measures used),^7 34^ and several population-based studies have identified significantly higher rates of dementia diagnoses in schizophrenia.^1 3–5 35 36^ These findings persist when controlling for dementia risk factors (eg, smoking, diabetes) and taking into account incentives to diagnose dementia (eg, obtaining admission to nursing homes). We lack a good understanding of the underlying cause of cognitive decline in schizophrenia.

Our systematic review found that published case–control studies have not found higher rates of dementia neuropathologies, specifically AD-related pathology, in cognitively impaired schizophrenia individuals compared with controls, or similar rates compared with patients with AD. How does this conclusion sit with the consistent finding of increased rates of dementia generally and AD specifically diagnosed in population studies?^1 35–37^ One potential explanation is that there is a neurodegenerative process intrinsic to schizophrenia, which is not detectable with standard neuropathological marker examination.^38^ For example, some abnormal neurodevelopmental processes (eg, relating to synaptic plasticity) may continue throughout life.^39^ Individuals with schizophrenia also have increased risk factors (eg, higher rates of drug and alcohol use, socioeconomic adversity and antipsychotic use) that impact cognition, which may not cause macroscopic pathological changes identifiable at postmortem examination. Another possible explanation is that normal ageing processes have a more notable effect on patients with schizophrenia due to diminished cognitive reserve, which has implications for the clinical assessment and diagnosis of dementia in these individuals.

Two studies did identify reduced Aβ42 in CSF of patients with schizophrenia compared with controls, but higher levels of Aβ42 compared with AD group. One possible explanation would be an early AD process; however, further studies would be needed to replicate this finding and to identify whether these patients do in fact go on to develop AD in the long term.

The cognitive assessment tools currently used to clinically diagnose dementia are potentially insufficiently sensitive to differentiate between dementia diseases and schizophrenia-related cognitive impairment. Positive and negative symptoms of schizophrenia could reduce scores on commonly used tests through distraction, reduced concentration, memory and fluency, so that patients with schizophrenia will score lower than the general population on cognitive testing, regardless of the age when they are tested. Given their lower starting point, even mild drops in test scores (for instance, associated with normal ageing), could place a patient below the cut-off used to diagnose dementia.

In addition to cognitive testing, assessments for dementia usually include brain imaging. Clinicians use MRI and CT to look for signs of vascular disease and hippocampal atrophy, which is suggestive of AD. The studies we identified that quantified hippocampal volume showed mixed results. It does seem that some patients with schizophrenia have reduced hippocampal volumes. However, the average age in these studies was lower than would be expected for AD and reduced hippocampal volume is consistently reported in patients with schizophrenia in the absence of diagnosed dementia, and early in the disease.^40–42^ It seems more likely that reduced hippocampal volume is a primary feature of the disease, rather than secondary to comorbid dementia. Reduced hippocampal volume may however increase diagnostic uncertainty when assessing a patient with schizophrenia for dementia, as it could mimic the appearance of hippocampal atrophy seen in AD.

Our analysis does not shed light on which of these non-mutually exclusive factors may be the primary driver of declining cognition in later life in schizophrenia. Further research on AD-related biomarkers such as PET and/or CSF amyloid and tau measures, markers of vascular dementia, PD, Lewy body dementia (LBD) and other measures of neurodegeneration (eg, neurofilament light chain) may help to confirm the true rate of AD and other dementias in the schizophrenia population. Future studies are also needed to further characterise the trajectory of cognitive decline in patients with schizophrenia who have been diagnosed with comorbid dementia, in comparison to the trajectory of normal ageing and dementia. A trajectory which appears distinct from either of these may be suggestive of a neurodegenerative process intrinsic to schizophrenia. Indeed, greater clinical use of cognitive assessments in the schizophrenia population would not only provide richer pools of data for research but may also better characterise patient functioning and needs.^7^

### Limitations

The higher rates of diagnosed dementia have been proposed to be related to the effects of vascular risk factors or antipsychotic medication.^43^ Only two studies in our review^19 44^ specifically looked for vascular pathology, and no studies looked specifically at medication-naive patients, which limits our ability to make conclusions about these potential dementia risk factors. In the wider literature, the picture for antipsychotic use is mixed, with some studies showing they *reduce* the risk of dementia,^5^ and that untreated psychosis is linked to worsening cognition.^45^ With regards to vascular pathology, the two studies included in our review did not find significantly higher rates than what would be expected in the general population, and population studies have also attempted to control for vascular risk factors. While these postmortem studies did not indicate higher rates of vascular pathology in cognitively impaired people with schizophrenia, we did not search for imaging studies which specifically identified vascular pathology; this is an interesting target for further review, as pure vascular dementia in the absence of AD pathology is not rare and may be an important cause of dementia in some individuals with schizophrenia.

Our meta-analysis findings were restricted to postmortem studies that examined evidence of AD (ie, NFTs and APs), rather than dementia more broadly. While this allowed us to reduce heterogeneity in the studies included for meta-analysis, it reduced the generalisability of the findings.

A number of studies that investigated postmortem changes in schizophrenia were not included in our study, as they did not specifically investigate patients who had established dementia or cognitive decline. Out of these, there were a handful of early studies that did in fact identify increased rates of AD in patients with schizophrenia based on neuropathological criteria.^46 47^ However, these studies were performed prior to the widespread use of formalised criteria required today. Following the advent of such criteria, these results were not replicated. In fact, older schizophrenia populations were found to have equal to or less pathology compared with normal elderly controls.

Our analysis was restricted to studies which examined patients who were reported to have dementia or had evidence of cognitive impairment. Our analysis was also limited to studies that included a comparison group. However, other postmortem studies that have looked at all patients with schizophrenia regardless of cognition have reported the same finding; no higher rates of AD pathology compared with that in the general population.^48–50^

Finally, the majority (14/17) of included postmortem studies were completed between 1990 and 2000, and before the publication of the National Institute for Ageing and the Alzheimer’s Association research framework which defined in vivo biological criteria for AD, which included measures of aggregated Aβ and tau in the CSF.^51^ Thus, the AD pathological assessments reported in the included studies do not fully align with current AD diagnostic criteria. The three studies in our review which analysed CSF for AD biomarkers did not report findings consistent with AD pathology, and none found any significant differences in total Tau and pTau between controls and patients with schizophrenia. Two studies did show lower levels of Aβ1–42 in patients with schizophrenia compared with controls, but these levels were still significantly higher than the levels seen in the AD comparison groups. In addition to Aβ and tau markers, we also searched for studies which included neurofilament light chain (NfL) in CSF as this is a non-specific marker of neurodegeneration. No studies were identified that used CSF NfL. However, we did not search for NfL in plasma. This may be an interesting avenue for further study.

Our review was not registered; however, no changes were made to our original protocol.

## Conclusion

Overall, we have shown that the observed increased rate of dementia in patients with schizophrenia is unlikely to be attributable to a higher rate of common neurodegenerative processes such as AD. Our findings highlight potential limitations in the aetiological diagnosis of dementia in older individuals with schizophrenia and the need for further investigation on what underlies the relatively modest reported declines in cognition with age (refer to papers included in the systematic review and are cited in the online supplemental file 2, table 1^52–63^).

10.1136/bmjment-2024-301017.supp1 Supplementary data

## Data Availability

Data are available upon reasonable request. Data extraction files from papers are available on request to the corresponding author.
