# Supplementary material for: Biomarkers of neurodegeneration in schizophrenia: systematic review and meta-analysis
Source: BMJ Ment Health. 2024 May 24;27(1):e301017. doi: 10.1136/bmjment-2024-301017 (PMC11129036; doi:10.1136/bmjment-2024-301017)
Supplement: Supplementary data [file bmjment-2024-301017supp002.pdf]

Supplementary Table 1: Characteristics and Findings of included studies

Author's Surname and Year	Pathology	vs AD	vs C	No. of Participants	Average Ages	Percentage Female	Cognitive Scoring	Inclusion in meta-analysis	Study Quality
Post Mortem Studies									
Arnold 1994 [53]	AP, NFT	↓ AP<br>↓ NFTs	↔ AP<br>↔ NFTs	Sz: 15<br>AD: 5<br>C:5	Sz: 76.9<br>AD: 79.0<br>C: 71.4	Sz: 40<br>AD:20<br>C: 60	Clinical Assessment	Y	Medium
Arnold 1996 [54]	NFT, GFAP, VIM	↓NFTs	↔ NFTs	Sz: 21<br>AD: 5<br>C: 12	Sz: 73.7<br>Sz (D): 82.2<br>AD: 77.6<br>C: 74.7	Sz: 57.1<br>AD: 40<br>C: 58	MMSE + Clinical Assessment	Y	Medium
Arnold 1998 [55]	AP, NFT, ✝	↓AP<br>↓NFTs	↔ AP<br>↔ NFTs	Sz: 23<br>AD: 10<br>C: 14	Sz: 79.8<br>AD: 81.8<br>C: 75.3	Sz: 65<br>AD: 50<br>C:57	MMSE<br>Sz: 12.3	Y	High
Casanova 1993 [56]	AP, NFT	↓AP<br>↓NFTs	↔ AP<br>↔ NFTs	Sz: 10<br>AD: 10<br>C: 10	Sz: 66<br>AD: 62<br>C(D): 61	Sz: 60<br>AD: 50<br>C(D): 30	Clinical Assessment	N (no standard deviations included)	Medium
El-Mallakh 1991 [57]	AP, nbM	↓ Frontal AP<br>↑ nbM	↔ Frontal AP<br>↔ Hipp. AP<br>↔ nbM	Sz: 10*<br>AD: 3<br>C: 10	Sz:84.2<br>AD: 68.3<br>C 72.6		Clinical Assessment	Y	High
Falke 2000 [58]	NFT, GFAPa, CD68, dN	↓NFTs	↔NFTs<br>↔GFAPa<br>↔CD68	Sz: 11<br>C:11<br>AD:12	Sz: 80.5<br>AD: 79.4<br>NC: 77.6	Sz: 75<br>AD: 83<br>NC: 36	Clinical Assessment	Y	High

			↔dN						
Gabriel 1996 [59]	SST, NPY CRH, VIP, CCK	↓VIP (2 regions)	↓SST (4 regions)<br>↓ CCK (3 regions)<br>↓NPY(2 regions)<br>↓ CRH (1 region)	Sz: 19<br>AD: 13<br>C: 8	Sz: 75.8<br>AD: 71.0<br>C: 82.6	Sz: 47<br>AD: 23<br>C: 78	CDR:<br>Sz: 2.16<br>AD: 4.45<br>C: 0.94	N (pathology did not include APs or NFTs)	Medium
Gabriel 1997 [60]	AP, PPD	↓AP	↔AP<br>↑PPD (in anterior cingulate cortex)	Sz: 19<br>AD: 24<br>C: 16	Sz: 74.1<br>AD: 73.6<br>C: 81.4	Sz: 42<br>AD: 33<br>C: 63	CDR:<br>Sz: 2<br>AD: 4.2<br>C: 1.2	Y	Medium
Haroutunian 1994 [20]	ChAT, AChE	↓ChAT<br>↓AChE	↔ChAT<br>↔AChE	Sz: 19<br>AD: 16<br>C: 9	Sz: 75.8<br>AD: 71.0<br>C: 82.5	Sz: 53<br>AD: 31<br>C: 78	CDR:<br>Sz: 2.53<br>AD: 4.45	N (pathology did not include APs or NFTs)	Medium
Niizato 1996 [22]	T+		↔T+	Sz: 7<br>C: 7	Sz: 63.4<br>C: 62.4	Sz:43<br>C: 0	Clinical Assessment	Y	Medium
Niizato 1998 [61]	AP, NFT		↔ AP<br>↔ NFTs	Sz:12<br>C: 12	Sz: 82.7<br>C: 83.3	Sz: 42<br>C:58	CDR:<br>Sz: 0.71	Y	Medium

Nishioka 2004 [62]	AP, NFT, Ast, 8-OHdG, Ki-67		↔ AP<br>↔ NFTs<br>↔ Ast	Sz: 10<br>C: 13	Sz: 75.2<br>C: 75.5	Sz: 70<br>C:69	MMSE<br>Sz: "Mod - Severe dementia"	Y	High
Powchik 1993 [21]	Alz-50	↓Alz-50	↔Alz-50	Sz: 9<br>AD: 13<br>C: 13	Sz: 79.1<br>AD: 78.7<br>C: 79.2		CDR:<br>All patients scored > 1	N (pathology did not include APs or NFTs)	Medium
Purohit 1993 [44]	AP, NFT, ✝✝**	↓AP	↔AP	Sz: 13<br>AD: 12<br>C: 12	Sz: 79.4<br>AD: 77.1<br>C: 75.3	Sz: 46	CDR:<br>Sz: 2.46	N (No quoted mean values or standard deviation. Descriptive findings of NFTs and AP	Medium
Purohit 1998 [45]	AP, NFT, ✝✝✝***		↔AP (PC and C)<br>↔ NFTs (PC and C)	Sz: 100<br>PC: 47<br>C:50	Sz: 78.5<br>PC: 76.9<br>C: 76.5		CDR:<br>Sz: 2.33	Y	Medium
Religa 2003 [63]	AP, Aβ, Aβ40, Aβ42	↓AP<br>↓Aβ	↔Aβ<br>↔Aβ40<br>↔Aβ42 (sz w/o Alz)<br>↑Aβ42 (sz w Alz)	Sz: 33<br>AD: 10<br>C: 11	Sz: 70.3<br>AD 81.6<br>C 82.8	Sz: 36<br>AD:70<br>HC: 82	CDR:<br>Sz (w/o : 1.96)<br>Sz (w alz): 2.14<br>AD: 4.10<br>C: 0.25	N (schizophrenia group was divided into subgroups with and without Alzheimer's)	Medium
Religa 2006 [64]	AP, NFT, Zn, Cu, Fe, Mn, Al, Aβ	↓ Zn<br>↓NFTs ****<br>↔Fe, Mn,	↔Zn<br>↔ NFTs ****<br>↔Fe, Mn, Al	Sz: 34<br>AD: 10<br>C: 14	Sz: 70.3<br>AD: 81.6<br>C: 82.8	Sz:31<br>Sz(A): 63<br>AD: 70<br>C: 86	CDR:<br>Sz: 1.85<br>Sz (A): 2.25<br>AD: 4.1<br>C: 0.45	N (schizophrenia group was divided into subgroups with and without presence of	Medium

		Al						amyloid,	
Hippocampal Volume Studies									
Huang 2020 [27]	HV		↓HV TRSz<br>↔HV NTRSz	Sz: 86<br>C: 53	TRSz: 48.5 ± 8.9<br>NTRSz: 46.5 ± 10.6<br>C: 44.8 ± 9.4	TRSz: 35<br>NTRSz: 35<br>C:0.49	MCCB<br>TRSz: 40.4<br>NTRSz: 45.4<br>C: 57.8	N (pathology did not include APs or NFTs)	Medium
Prestia 2011 [31]	HV	↔HV	↓HV	Sz: 20<br>AD: 20<br>C: 19	Sz: 67.4<br>AD: 72.7<br>C: 72.5	Sz: 50<br>AD: 60<br>C: 68	MMSE<br>Sz: 22.8<br>AD: 22.0<br>C: 29.1	N (pathology did not include APs or NFTs)	Medium
Pujol 2014 [28]	HV		↔HV*****	Sz: 51<br>C: 49	Sz: 48.9<br>C:52.8	Sz: 45<br>C:47	WAIS-III-R<br>RAVLT<br>MMSE (spanish version)	N (pathology did not include APs or NFTs)	Medium
Rivas 2023 [30]	HV		↓HV Sz(D)<br>↔HV Sz	Sz (D): 10<br>Sz: 10<br>C: 15	Sz(D): 69.5,<br>Sz: 58.0<br>C: 60.0	All participants: 54.2	HVLT, RCFT,<br>FCSRT,<br>BNT: Sz and Sz (D) ↓ scores vs C	N (pathology did not include APs or NFTs)	Medium

Sachdev 2000 [29]	HV		↔HV<br>↓ L sided AHV	Sz: 20<br>C:24	Sz: 64.4<br>C:72.7	Sz: 40<br>C:21	MMSE<br>Sz: 25.8<br>C: 29.4	N (pathology did not include APs or NFTs)	Medium
Amyloid / Tau measures detected in CSF									
Albertini 2012 [24]	T-tau, P-tau, A**β**42, other A**β** peptides	↑ A**β**142<br>↓ T-tau<br>↓ P-tau	↓ A**β**42 ******<br>↔T-tau<br>↔P-tau	Sz: 11<br>AD: 20<br>C: 20	Sz: 69.2<br>AD: 70.6<br>C: 65.3	Sz: 72.7<br>AD: 60<br>C: 30	MMSE<br>Sz: 22.5<br>AD: 19.7<br>C: 28.9	N (pathology did not include APs or NFTs)	Medium
Frisoni 2011 [25]	T-tau, P-tau, A**β**42	↑A**β**42<br>↓ T-tau<br>↓ P-tau	↓A**β**42<br>↔T-tau<br>↔P-tau	Sz: 11<br>AD: 20<br>C:26	Sz: 69.2<br>AD: 71.1<br>C: 61.3	Sz: 73<br>AD: 75<br>C: 17	MMSE<br>Sz: 22.1<br>AD: 21.3<br>C: 29.3	N (pathology did not include APs or NFTs)	Medium
Schönknecht 2003 [26]	T-tau, P-tau		↔T-tau<br>↔P-tau	Sz: 19<br>C: 20	Sz: 64.2 (group > 50)<br>C:65.5 (group > 50)	Sz: 67<br>C:60	MMSE<br>Sz: 27.7<br>C: 29.5	N (pathology did not include APs or NFTs)	Medium

Quality assessment was performed using a modified version of the NIH Quality Assessment Tool for Observational Cohort and cross sectional studies. Studies were given a score of between 0 and 12, with 0-4 classed as low, 5-8 classed as medium, and 9-12 classed as High.

↔ No significant difference

↑ Significantly higher

↓ Significantly lower

✚ Lewy bodies, ubiquinated dystrophic neurites, GFAP in astrocytes, resting and active microglia

✝✝ Cerebral Amyloid Angiopathy, Lewy bodies, vascular pathology such as infarcts, atherosclerosis, arteriolar sclerosis, Features of Parkinson's disease, diffuse Lewybody disease, Creutzfeldt-Jakob disease, and Pick's disease.

✝✝✝ Cerebral Amyloid Angiopathy, Lewy bodies, vascular pathology such as infarcts, atherosclerosis, arteriolar sclerosis, neocortical neuronal loss, hirano bodies, neuropil degeneration and gliosis; hippocampal degeneration, granulovacuolar degeneration

* Study also included 7 Schizophrenia patients without cognitive impairment, which we have not included
** No evidence of morphological features of other dementing neurodegenerative conditions, such as multi-infarct dementia, Parkinson's disease, diffuse Lewy-body disease, Creutzfeldt-Jakob disease, or Pick's disease, was found
***Postmortem neuropathologic examination findings: Alzheimer's disease: 9, Parkinson's disease: 2, Multi infarct dementia: 1, Multiple sclerosis: 1, Ischaemic cerebrovascular disease 23, Secondary neoplasms 3, Brainstem Haemorraghe: 1, parietal lobe haemangioma: 1, Frontal Leukotomy: 11
**** NFTs were only present in samples from the AD group
*****Steeper decline as function of age in Sz group
****** Several other A**β** peptides also lower

Key:

8-OHdG	8-hydroxy-2' -deoxyguanosine
AChE	Acetylcholinesterase activity
AD	Alzheimer's disease
AHV	Amygdala-hippocampal volume
Al	Aluminium
Alz-50	Alz-50 immunoreactivity
AP	Amyloid plaque
Ast	Astrocytosis
Aβ	Total amyloid β-peptide
Aβ40	Amyloid β-peptide-40
Aβ42	Amyloid β-peptide-42

BNT	Boston Naming Test
C	Controls
CA	Clinical Assessment, including chart review, collateral history, direct clinical observation
CCK	Cholecystokinin
CD68	CD68 microglia,
CDR	Clinical Dementia Rating
ChAT	Choline acetyltransferase activity
CRH	Corticotropin releasing hormone
Cu	Copper
dN	Density of neurons
FCSRT	Free and Cued Selective Reminding Test
Fe	Iron
GFAP	Glial fibrillary acidic protein
GFAPa	GFAP +ve astrocytes,
Hipp.	Hippocampal
HV	Hippocampal Volume
HVLT	Hopkins Verbal Learning Test
Ki-67	Antigen KI-67
MCCB	MATRICS™ Consensus Cognitive Battery
MMSE	Mini mental state exam
Mn	Manganese

nbM	No. of cells in the nucleus basalis of Meynert
NFT	neurofibrillary tangle
NPY	Neuropeptide y,
nTRSz	Non-treatment Resistant Schizophrenia
P-tau	Phosphorylated Tau
PC	Other psychiatric controls
PPD	Presynaptic protein density (synaptophysin, syntaxin, synaptosomal associated protein-25-kd)
RAVLT	Rey Auditory Verbal Learning Test
RCFT	Rey Complex Figure Test
SST	Somatostatin
Sz	Schizophrenia
sz w/ Alz	Schizophrenia group with alzheimer's pathology
sz w/o Alz	Schizophrenia group without alzheimer's pathology
T+	Tau +ve Neurons
T-tau	Total Tau
TRSz	Treatment Resistant Schizophrenia
VIM	Vimentin
VIP	Vasoactive intestinal polypeptide
WAIS	Wechsler Adult Intelligence Scale
Zn	Zinc
